# Knowledge, Perceived Importance, Current Uptake, and Willingness to Adopt Healthy Sustainable Dietary Actions: A Cross-Sectional Study of UK Adults

**DOI:** 10.3390/nu18030534

**Published:** 2026-02-05

**Authors:** Danielle J. Guy, Jeffery Bray, Katherine M. Appleton

**Affiliations:** 1 Department of Psychology, Faculty of Media, Science and Technology, Bournemouth University, Poole BH12 5BB, UK; k.appleton@bournemouth.ac.uk; 2 Bournemouth University Business School, Bournemouth University, Poole BH12 5BB, UK; jbray@bournemouth.ac.uk

**Keywords:** sustainability, healthy eating, knowledge, perceived importance, perceived value, willingness, dietary change

## Abstract

**Background/Objectives**: Sustainable diets are essential for public health, food system resilience, and environmental protection, yet engagement with healthy sustainable dietary actions is varied. This study investigated knowledge, perceived importance, current uptake, and willingness to adopt healthy sustainable dietary actions among the general UK population. **Methods**: A cross-sectional study was conducted using a self-report questionnaire completed by 635 adults (205 males and 430 females; mean (SD) age = 43 (16.8) years) in the UK. **Results**: Considerable variation in knowledge, perceived importance, and current engagement was found across the dietary actions investigated. All three were highest for familiar, health-aligned behaviours, while actions with a stronger environmental focus that were less conventional or culturally unfamiliar were less well understood, perceived as less important, and less often engaged with. Willingness to adopt actions not currently practised was most strongly predicted by perceived importance (smallest β = 1.21, *p* < 0.001), with perceived value also significant for several actions (smallest β = 0.86, *p* < 0.001). Knowledge and perceived impact were generally non-significant. Demographic and lifestyle factors showed smaller effects, with greater willingness among younger individuals (smallest β = −0.24, *p* = 0.01) and those with higher education (smallest β = 0.51, *p* = 0.01). **Conclusions**: These findings suggest some knowledge and engagement with healthy sustainable dietary actions in the UK. However, future campaigns may need to go beyond awareness-raising to emphasise the personal significance and value of these actions. These cognitive factors also showed broad applicability across demographic and lifestyle variables, suggesting potential for widely effective interventions.

## 1. Introduction

Healthy sustainable eating is essential for addressing public health, social, and environmental challenges associated with current food systems [1,2]. Specific actions, such as reducing food waste, adopting plant-based diets, and choosing seasonal or organic produce, represent behaviours that align with public health goals while reducing environmental impact [1,2,3,4]. These actions are important for improving population health, mitigating climate change, and ensuring future food security [1,2,3,4]. However, significant variability exists in the extent to which individuals engage in these behaviours, with some actions being more widely adopted, while others face greater resistance [1,4].

In order to enact any behaviour, one must first be aware of and have knowledge of it [5]. Awareness and knowledge of healthy sustainable eating have been shown to be important in these behaviours [5,6,7], but knowledge can be limited [7,8] and varies considerably across different dietary actions [7,8,9]. For instance, while behaviours like reducing food waste are well known, others, such as incorporating alternative protein sources, are less so [9]. As a result, rather than measure healthy sustainable eating as a single construct, examining knowledge at the level of specific actions, within the same population and using a consistent set of measures, may provide more nuanced insight into behavioural uptake.

Knowledge alone, furthermore, cannot guarantee future action [5]. A range of additional cognitive factors, including attitudes, motivation, and self-efficacy, have previously been investigated in relation to healthy sustainable eating to try and improve understanding [5,7,9,10,11]. Of these cognitive factors, perceived importance has been identified as crucial for sustainable or pro-environmental behaviours [5]. Perceived importance refers to a subjective judgement of the significance of something to an individual. When actions are viewed as personally important, they are more likely to be prioritised by individuals [12,13,14]. For example, individuals who value the health benefits of plant-based diets are more likely to increase their consumption of legumes [14], and those who recognise the environmental importance of reducing meat consumption are more inclined to engage in this behaviour [12]. Perceived importance has been identified as a primary determinant of healthy sustainable eating in several studies [11,15]. These findings align with behavioural theories emphasising motivational salience and value-based decision-making [15,16,17], suggesting that perceived importance may play a stronger role than information alone in shaping healthy sustainable dietary behaviour. Understanding the range of knowledge and the perceived importance of different sustainable dietary behaviours may provide insights into why certain actions are more widely adopted than others.

Additional cognitive variables of potential relevance for sustainable behaviours include environmental concern, perceived impact as a result of undertaking the behaviour, and a preference to undertake small changes [5,7,8]. Environmental concern relates to worry or concern over environmental issues, including climate change, biodiversity loss, habitat loss, and animal welfare [9,10,12]. Many studies report associations between these concerns and increased consumption of sustainable diets [9,10,12,13]. Perceived impact refers to the subjective sense of how one’s actions can influence social and environmental outcomes, and relates to both the belief that ‘my behaviour will make a difference’, and the belief that ‘my behaviour will make a difference even if only I undertake it’. Several studies now suggest associations between belief in likely benefit and the conduct of specific healthy sustainable actions [14], a lack of action in those who fail to recognise the links between diet and climate change, or the strength of these associations [7,12]. Repeated work further suggests a lack of action as a result of a sense of futility—the idea that actions will only have impact if undertaken at a collective level [7,8,13]. A lack of sustainable action has also been associated with a perceived need to make substantial changes for any impact. A preference for small changes has been identified specifically in relation to healthy sustainable eating [12], such that individuals may be willing to pay a bit more for outdoor-reared pork, but to stop eating pork is a step too far [12]. These additional cognitions may further aid understanding of the adoption of healthy and sustainable eating actions.

Current engagement in sustainable dietary behaviour varies considerably. Willingness to engage in sustainable dietary behaviours likely also varies widely, with potential influence from the same or similar underlying concerns. Moreover, barriers to undertaking sustainable action, such as cost, time constraints, and perceived effort, are often also reported. Individuals may aim to undertake sustainable behaviours but face challenges such as higher costs of local foods or organic products [18], limited time for meal planning [13], or the perceived inconvenience of changing established eating habits [14]. These structural barriers are frequently reported in assessments of wider healthy eating [19,20,21] and also typically cluster by demographic (e.g., age, income) and lifestyle characteristics (e.g., shopping habits, dining habits) [19,20,21]. As such, healthy sustainable dietary behaviour is shaped not only by cognitive factors but also by the broader social and material context in which food choices are made [5,6,11].

Taken together, existing research highlights the importance of motivational and cognitive factors in sustainable eating, yet many studies focus on single behaviours or broad dietary patterns [22,23,24]. Less is known about how knowledge, perceived importance, current uptake, and willingness to adopt differ across a wide range of specific healthy sustainable dietary actions within the same population. Addressing this gap may help explain why some actions are readily adopted while others remain resistant to change.

The present study, therefore, aims to systematically compare multiple healthy sustainable dietary actions within a UK sample, examining levels of knowledge, perceived importance, current uptake, and willingness to adopt each action. In addition, the study explores how cognitive factors, alongside demographic and lifestyle characteristics, are associated with both current engagement and willingness to adopt healthy sustainable dietary behaviours. The study is descriptive and exploratory in nature, aiming to compare patterns across actions rather than to establish causal relationships.

Given the heterogeneity of behaviours examined and the limited prior evidence directly comparing multiple sustainable dietary actions within a single population, the present study adopts a descriptive and exploratory framework. Rather than testing narrowly specified directional predictions, the aim is to examine comparative patterns across actions and identify consistent associations across behavioural domains.

Thus, the present study investigates seven hypotheses. It was hypothesised that participants will: (1) have more knowledge about some healthy sustainable dietary actions than others; (2) perceive some healthy sustainable dietary actions as more important than others; (3) currently engage in some healthy sustainable dietary actions more than others; and (4) that demographic, lifestyle, and cognitive factors will predict current engagement in healthy sustainable dietary actions. Additionally, it was hypothesised that (5) participants will be willing to undertake some healthy sustainable dietary actions more than others; (6) that participants will be willing to adopt some actions with which they are not currently engaging; and (7) that various demographic, lifestyle, and cognitive factors will predict willingness to adopt these actions.

Distinguishing between current uptake and willingness to adopt may help identify actions that represent realistic and acceptable targets for intervention. These actions may present opportunities for behaviour change. By jointly examining these constructs across multiple actions, this study seeks to identify potential leverage points for behaviour change and to clarify the relative importance of motivational versus informational factors in healthy sustainable eating. All hypotheses were specified before the data were collected.

## 2. Materials and Methods

### 2.1. Design

A self-report questionnaire study with a cross-sectional design was used.

### 2.2. Questionnaire

The full questionnaire can be found in the Supplementary Materials; it was split into four sections:

#### 2.2.1. Intake Frequency Estimate

The first section consisted of an adaptation of the Leeds Food Frequency Questionnaire [25], modified by the authors to align with the healthy sustainable dietary actions included in this study. This measure assessed the frequency of consumption of food items over the past month. Food groups selected were relevant to the aspects of healthy sustainable eating investigated. These were animal- and plant-based protein-rich foods, high-carbohydrate foods, fruits, and vegetables. The response choices available were ‘Everyday’, ‘3–5 times a week’, ‘1–2 times a week’, ‘1–2 times a fortnight, ‘Less than once a month’, and ‘Never’. Drinks were not included in the FFQ, as the measure focused specifically on food items aligned with the healthy sustainable dietary actions assessed in this study.

#### 2.2.2. Sustainable Actions

**Figure 1:**
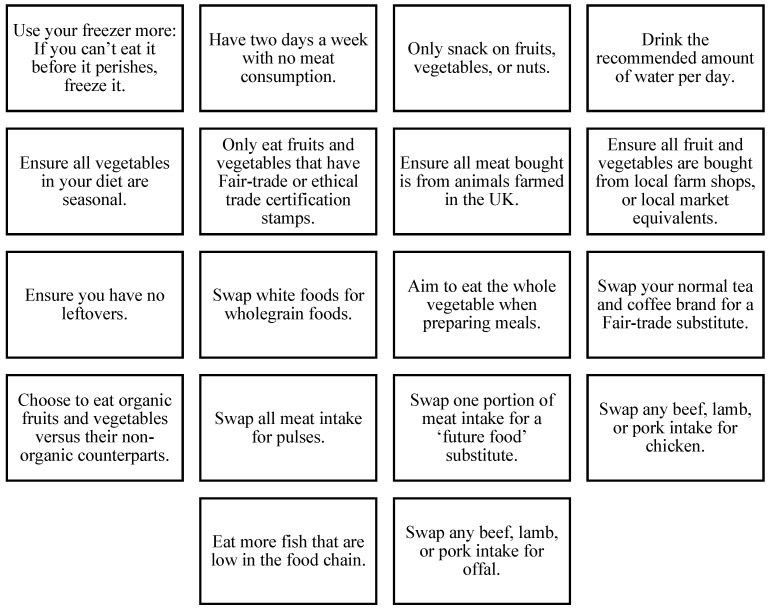
The eighteen sustainable dietary actions used in our questionnaire. Please note that ‘future foods’ refer to land-efficient, nutrient-rich alternatives to traditional animal-sourced foods, such as crickets, larvae, and mealworms, that can be produced at scale with key environmental benefits [27].

Section two consisted of twenty-three potential dietary changes, of which eighteen were identified as healthy sustainable dietary actions, as defined by Guy et al. [26]. These actions are presented in Figure 1. All actions were selected for their potential to enhance both environmental sustainability and personal health outcomes [1], although they represent a subset of the broader spectrum of possible solutions. Environmental sustainability in this study refers specifically to greenhouse gas emissions, land use, and water use, which formed the basis for selecting these actions. All actions were deliberately selected to represent small, incremental dietary changes that could plausibly appeal to a wide range of individuals, regardless of their current dietary patterns or motivations. Rather than focusing on larger dietary shifts, the actions were framed as modest substitutions, frequency reductions, or purchasing and preparation choices that may be perceived as achievable within everyday eating practices. This approach was informed by previous evidence suggesting that preferences for small changes can facilitate engagement with healthy sustainable eating, particularly among individuals who may be resistant to more transformative dietary recommendations [12].

Despite this shared focus on relatively small, actionable dietary changes, the selected actions differ in their practical characteristics and behavioural requirements. This heterogeneity reflects the breadth of behaviours commonly discussed in relation to healthy sustainable diets and was retained to allow comparison across a wide range of specific actions. Differences in knowledge, perceived importance, current uptake, and willingness to adopt were, therefore, examined across all actions.

Five additional actions that we did not classify as healthy and/or sustainable were also included as distractors [28]. These were ‘eat more tropical fruits’, ‘only consume fish that are high in the food chain’, ‘only eat non-free-range eggs’, ‘discard food when it has reached its best-before date, even if it is still safe to consume’, and ‘eat more beef than chicken’. These distractors were selected to reflect common misconceptions or intuitively appealing but environmentally unfavourable practices, thereby increasing the discriminative validity of the knowledge assessment.

Actions were presented in a randomised order, and participants were asked to identify all those that they believed were ‘healthy sustainable actions’. This task assessed perceived knowledge only; it did not measure current behaviour or willingness. Knowledge was operationalised as recognition of actions commonly promoted as healthy and sustainable, reflecting applied, everyday understanding rather than detailed scientific knowledge. All responses were subsequently ranked based on the number of participants selecting them to represent knowledge of the whole sample; higher ranks represented the actions most selected. Importantly, the actions were treated as individual behavioural options rather than as indicators of a single latent construct. The aim was not to model healthy sustainable eating as a unitary behaviour but to compare patterns across multiple specific actions that share a common framing as small, healthy, and sustainable relevant changes.

Next, participants were asked to rate each of the 18 sustainable actions on: how important they believed each action to be for achieving a healthy sustainable diet (response options: ‘Very important’, ‘Somewhat important’, ‘Neutral’, ‘Somewhat unimportant’, ‘Not at all important’); their current uptake for each action (response options: ‘Everyday’, ‘3–5 times a week’, ‘1–2 times a week’, ‘1–2 times a fortnight’, ‘Less than once a month’, ‘Never’), and how willing they would be to carry out each action going forward (response options: ‘Very likely’, ‘Likely’, ‘Neutral’, ‘Unlikely’, ‘Very unlikely’, ‘N/A’).

#### 2.2.3. Healthy Sustainable Eating

Section three assessed six broad concepts around healthy sustainable eating, as gained from earlier work [3,21]. These were **knowledge** (‘I know what a healthy diet consists of’, ‘I know what a sustainable diet consists of’); **impact** (‘I understand the impact my diet has on my health’, ‘I understand the impact my diet has on the environment’); **perceived importance** (‘I think sustainability is important’, ‘I think it is important to live a lifestyle that is sustainable’); **environmental concern** (‘I care about the environment’, ‘I think it is important to live a lifestyle that is environmentally friendly’); **small changes** (‘I am willing to make a small change to make my diet more sustainable’, ‘I think making a big change to make my diet more sustainable would be unachievable (-)’); **perceived value** (‘My dietary actions alone can make a difference’, ‘Everyone needs to eat more sustainably for this to make a difference (-)’). Response options for each statement were ‘Strongly agree’, ‘Agree’, ‘Neither agree nor disagree’, ‘Disagree’ and ‘Strongly disagree’.

Internal reliability was assessed using Cronbach’s alpha. Environmental concern (α = 0.79) and perceived importance (α = 0.77) demonstrated good internal consistency. Knowledge (α = 0.54), impact (α = 0.49), and small changes (α = 0.44) demonstrated moderate to low internal consistency, while perceived value showed low reliability (α = 0.15). These lower reliability values likely reflect the use of brief two-item scales capturing conceptually broad constructs and should be interpreted with appropriate caution.

#### 2.2.4. Demographic and Lifestyle Characteristics

Section four asked questions on demographic characteristics: age (7–10 year pre-defined categories); gender (‘male’, ‘female’, ‘non-binary’, ‘prefer not to say’); education (eight options taken from the UK Office for National Statistics [29]); employment status (nine options from the UK National Statistics Socio-economical Classifications (NS-SEC) [30]); household income (six categories informed by UK government definitions of income brackets relevant to tax thresholds [31]) and a measure of social deprivation (based on household size and number of bathrooms, adapted from the UK Census 2021 [32]). Lifestyle questions were: frequency of eating with others (‘Never’, ‘Sometimes’, ‘About half the time’, ‘Most of the time’, ‘Always’), and frequency of food cooking (‘Never’, ‘1–2 times a week’, ‘3–4 times a week’, ‘5–6 times a week’, ‘Every day’).

### 2.3. Questionnaire Administration

Online and paper-based completion was enabled, ensuring the questionnaire was inclusive for the general population. The questionnaire took no longer than thirty minutes to complete. Participants were recruited across the UK from research pools, personal contacts, social media, businesses, and Bournemouth University students and staff to ensure diverse demographic and lifestyle representation. Participants were eligible if they were aged over 18 years and residing in the UK. Participants were not made aware of the true purpose of the study, and to reduce demand characteristics; recruitment flyers and information sheets promoted the study as investigating ‘Dietary patterns: Public perceptions and understanding’. The study ran for twelve months (March 2022–February 2023), with an aim of recruiting 50 respondents per month, to ensure that all seasons were equally considered [33]. Psychology participants from Bournemouth University received 0.5 credits. No other compensation was given.

Prior to roll-out, 10 participants (mean age = 40.5 years; 6 male, 4 female) were asked to complete the questionnaire and verbalise thoughts that emerged whilst doing so to ensure the intended interpretation. Only formatting changes were suggested by participants, and all of these were implemented. Following this, the validity of the current uptake questions (that is, the self-reported frequency of performing each healthy sustainable dietary action) was confirmed in a pilot study. Sixty-five participants (mean age = 41.3 years, 49% male; 51% female) completed a food diary for 4 consecutive days before completing the questionnaire one week later. For the food diaries, participants were asked to record all foods and drinks consumed in as much detail as possible. Participants were asked if they discarded any food that day, and if they stored any food items for future use. Participants were given training on completing the food diary, and once the participant had completed the first day of food diaries, the lead researcher (DJG) checked the diary for likely completion and answered any questions from participants. Following completion of both the food diaries and questionnaire, participants’ food diaries were compared with their questionnaire responses to evaluate the accuracy and reliability of the self-reported uptake of sustainable dietary actions. Visual inspection revealed >70% agreement between recorded dietary behaviours and questionnaire responses, indicating acceptable agreement consistent with previous validation studies of brief dietary assessment tools, supporting the use of these measures for comparative and exploratory analyses [34,35].

The pilot study served as the initial validation of the sustainable actions that were later implemented in the main study. Additional validation using the data from the main study was also undertaken. Reported engagement in some sustainable actions was again compared with dietary intakes as reported in the Food Frequency Questionnaire, confirming that these sustainable actions were reflective of actual behaviour. Results showed that participants were consistent in their reporting, with agreement again exceeding 70% (see Supplementary Materials, Tables S1 and S2).

### 2.4. Data Analyses

Data were first screened for missing values. If responses were missing by more than 20% for a particular participant, all data for this individual were discarded. If 80% or more were completed, missing values were imputed, with neutral points, or the average score where more than one response was selected. The characteristics of the remaining participants were assessed through descriptive statistics. No differences were found between the data sets for the pilot study and the main study, so they were included as one for analysis. Demographic information was first examined using χ^2^ tests to investigate the representative nature of the sample for the UK population, in terms of gender and age, according to the UK Census 2021 [29].

To examine knowledge of different healthy sustainable actions, actions were ranked based on the number of participants identifying them as healthy and sustainable. The perceived importance of each sustainable action was scored from −2 to +2, where higher values indicated greater perceived importance, and averaged across participants. One-sample *t*-tests were performed to assess whether importance ratings differed significantly from the neutral midpoint of the scale for each action. Paired *t*-tests were then used to compare actions pairwise in order to establish relative ordering. Actions that did not significantly differ from one another (*p* > 0.05) were grouped and assigned the same rank. These ranks were subsequently used to define the colour gradients in the heat map visualisation, with darker shades representing higher-ranked actions and lighter shades representing lower-ranked actions. This approach enabled visual comparison of relative patterns across actions while avoiding overinterpretation of small numerical differences.

Current uptake of sustainable actions was assessed by converting categorical frequency responses into ‘number of times per week’ based on category medians. All uptake values, therefore, represent estimated frequencies of action performance per week. Actions were ranked based on mean weekly frequency using paired-samples comparisons, with actions that did not differ significantly grouped together. These uptake ranks were subsequently used to determine heat map colour intensity. Multiple linear regression analyses were also conducted to explore demographic, lifestyle, and cognitive factors associated with current engagement.

Willingness to undertake each healthy sustainable action was analysed in the same manner as for perceived importance. Mean willingness scores were compared using paired samples *t*-tests to generate ranked grouping based on non-significant differences, and these ranks were used to define the colour gradients displayed in the heat map.

Finally, a NCU-W score was created for each sustainable action per participant by reverse-scoring the current uptake values to represent the degree to which an action was not currently undertaken (in number of days per week), and combining this with the willingness score (−2 to +2), to demonstrate the degree to which each participant was **N**ot **C**urrently **U**ndertaking an action but **W**illing to undertake it (**NCU-W**). The resulting NCU-W score is a composite behavioural opportunity index rather than a direct measure of behaviour or intention. Scores ranged from −14 (I am not currently doing this at all (7) × I am not willing to do this at all (−2)) to +14 (I am not currently doing this at all (7) × I am very willing to do this (+2)), with scores in between representing the following: I do this a little (5.5) and I am willing (1) or not willing (−1) to do this a little. For example, an individual who does not currently perform an action at all but reports high willingness to adopt it would receive a high positive NCU-W score, indicating substantial opportunity for future behaviour change. Higher positive values indicate actions that are infrequently performed but associated with high willingness to adopt, whereas negative values indicate frequent current engagement or low willingness to change. By integrating both behavioural absence and motivational readiness, the NCU-W score measure captures opportunities for change that would not be identified by willingness ratings alone. One-sample *t*-tests were performed to examine differences in NCU-W scores. Paired samples *t*-tests were then used to generate ranked groupings based on non-significant differences, and these ranks were used to define heat map colour intensity for NCU-W scores. Multiple linear regression analyses were again conducted to examine the demographic, lifestyle, and cognitive factors that were associated with willingness to adopt high-opportunity sustainable actions.

All regression models were conducted using the enter method. Correlational analyses were performed prior to regression modelling to assess for multicollinearity and confirm model integrity. At this stage, high correlations were found between scales for ‘perceived importance’ and ‘environmental concern’; thus, responses for the two scales were combined into one ‘perceived importance’ scale. All statistical analyses were conducted using IBM SPSS Statistics (version 28) and Microsoft Excel. Significance was set at *p* < 0.05.

## 3. Results

### 3.1. Study Population

A total of 678 participants completed the questionnaire, and of these, 43 were excluded based on not meeting the inclusion criteria and/or the number of missing values. Questionnaires were completed evenly across the year (summer: 173 responses; autumn: 169 responses; winter: 170 responses; spring: 171 responses). Demographic information of the 635 included respondents can be found in Table 1.

Based on the 2021 UK Census, for gender, males were underrepresented (χ^2^(1) = 11.55, *p* < 0.001) and females were overrepresented in the study; for age, the sample was representative (χ^2^(5) = 8.57, *p* < 0.001).

### 3.2. Knowledge, Perceived Importance, Current Uptake, Willingness, and NCU-W Across Healthy Sustainable Actions

An integrated summary of participants’ knowledge, perceived importance, current uptake, willingness to adopt, and NCU-W scores across all healthy sustainable dietary actions is presented in Table 2. This table allows for direct comparison of relative patterns across constructs for each action. Cells are colour-shaded using a heat map scale to indicate relative magnitude within each construct, with darker shading representing higher values and lighter shading representing lower values. This visual encoding is intended to facilitate comparison of patterns across actions and constructs.

#### 3.2.1. Knowledge of Healthy Sustainable Dietary Actions

Overall knowledge of healthy sustainable actions was high, although substantial variation was observed across individual actions, as shown in Table 2 (Knowledge column). Knowledge of sustainable dietary actions differed significantly across actions (overall omnibus test, Cochran’s Q(22) = 5705.88, *p* < 0.001).

Actions aligned with familiar health-related behaviours were recognised by the largest proportions of participants, including using the freezer to reduce food waste (94%), having two meat-free days per week (79%), snacking on fruits and vegetables (78%), and drinking the recommended amount of water (72%). In contrast, less conventional but environmentally impactful actions were recognised by substantially fewer participants, including substituting meat with future food alternatives (25%), consuming fish lower in the food chain (30%), and replacing ruminant meat with offal (6%).

In addition to healthy sustainable actions, several non-sustainable ‘distractor’ items were included to assess recognition accuracy. A proportion of respondents incorrectly identified these actions as healthy and sustainable. Specifically, 13% selected ‘eat more tropical fruits’ and 11% selected ‘only consume fish that are high in the food chain’, despite these being environmentally unfavourable practices. Smaller proportions also incorrectly endorsed ‘only eat non-free-range eggs’ (5%), ‘discard food at the best-before date even if it is still safe to consume’ (4%), and ‘eat more beef than chicken’ (2%). Notably, some distractor actions were recognised more frequently than genuinely sustainable but less familiar actions, such as swapping ruminant meat for offal (6%).

#### 3.2.2. Perceived Importance of Healthy Sustainable Dietary Actions

Mean perceived importance ratings for each action are reported in Table 2 (Perceived Importance column). Ratings differed significantly across actions (overall omnibus test, F(12.31, 7802.85) = 163.32, *p* < 0.001), demonstrating variation in the relative importance participants assigned to specific actions. All actions were perceived as important (significantly more positive than neutral), with exceptions for actions ‘Swap one portion of meat intake for a “future food” substitute’ and ‘Swap any beef, lamb, or pork intake for offal’, which were perceived as unimportant. These actions had a mean importance of scores of −0.31 and −0.45 respectively.

#### 3.2.3. Current Uptake of Healthy Sustainable Dietary Actions

Average weekly uptake of each healthy sustainable dietary action is summarised in Table 2 (Current Uptake column). Weekly uptake frequency differed significantly across actions (Friedman’s χ^2^(17) = 3541.94, *p* < 0.001, Kendall’s W = 0.33), indicating a moderate-to-large effect. Routine and convenience-based actions, such as drinking the recommended amount of water (M = 4.29 times/week), ensuring you have no leftovers (M = 4.11 times/week), and using the freezer more (M = 4.00 times/week), were performed most frequently. In contrast, actions involving less familiar dietary substitutions, including eating fish lower in the food chain (M = 0.46 times/week), future food consumption (M = 0.18 times/week), and offal consumption (M = 0.14 times/week) were least frequently undertaken. With the exception of the ‘two meat-free days per week’ action (which was structured as a weekly target rather than a daily behaviour), all actions could theoretically be undertaken up to seven times per week. This classification allowed consistency in frequency reporting while accurately reflecting adherence to the behavioural targets.

All regression models included the same set of predictors to allow direct comparison of associations across different dietary actions, rather than to optimise prediction for any single outcome. Current engagement in sustainable dietary actions was predicted by the demographic, lifestyle, and cognitive factors included in our regression models (detailed coefficients are reported in Supplementary Materials, Table S3). Across actions, adjusted R^2^ values ranged from 0.03 to 0.16. Age, educational attainment, and involvement in food cooking were statistically significant predictors for at least one action (standardised βs range from 0.10 to 0.30, *p* < 0.05), although the presence and direction of effects varied across actions. Among the cognitive variables, perceived importance was statistically significantly associated with current uptake for most actions (15 of 18 models), with large and consistent effect sizes (standardised βs range from 0.69 to 1.40, *p* < 0.01). Knowledge was also significantly associated with uptake for several actions (standardised βs range from 0.21 to 0.57, *p* < 0.05), but was non-significant for others. Perceived impact, perceived value of undertaking the behaviour, and willingness to make small changes were statistically significant predictors for a smaller subset of actions, with generally lower standardised coefficients.

Across models, explained variance was modest (adjusted R^2^ values ranging from ~0.03 to ~0.18), indicating that while the included demographic, lifestyle, and cognitive variables contribute meaningfully to understanding engagement in specific actions, a substantial proportion of variance remains unexplained.

#### 3.2.4. Willingness to Adopt Healthy Sustainable Dietary Actions

Participants’ willingness to adopt healthy sustainable dietary actions is presented in Table 2 (willingness to adopt column). Mean willingness ratings differed significantly across actions (overall omnibus test, F(12.46, 5857.50) = 204.16, *p* < 0.001). Respondents were willing to adopt most actions (mean scores significantly above neutral), with the exception of ‘Swap one portion of meat intake for a “future food” substitute’ (M = −0.91) and ‘Swap any beef, lamb, or pork intake for offal’ (M = −0.95), which were rated negatively. Participants expressed the greatest willingness to adopt routine and convenience-based actions, including ‘using the freezer more’ (M = 1.58), ‘drinking the recommended amount of water per day’ (M = 1.37), and ‘ensuring no leftovers’ (M = 1.34).

#### 3.2.5. NCU-W Score (Not Currently Undertaking but Willing to Do)

Composite NCU-W scores are reported in Table 2 (NCU-W column). The NCU-W score refers to participants indicating that they were **N**ot **C**urrently **U**ndertaking the action but were **W**illing to do so. NCU-W values reflect the product of reversed uptake (0–7 days/week not undertaken) and willingness (−2 to +2), yielding a theoretical range of −14 to +14; higher positive values indicate lower current uptake combined with higher willingness to adopt. NCU-W scores differed significantly across actions (Friedman’s χ^2^(17) = 3735.10, *p* < 0.001, Kendall’s W = 0.35), indicating a moderate-to-large effect. The highest opportunity scores were observed for using the freezer more, increasing water intake, avoiding leftovers, swapping white foods for wholegrains, and adopting two meat-free days per week. In contrast, negative NCU-W scores were observed for future food substitution, fish lower in the food chain, and offal consumption.

Multiple linear regression results are shown in the Supplementary Materials, Table S4. Across models, adjusted R^2^ values ranged from 0.05 to 0.19, indicating modest explanatory power. Age was a statistically significant predictor of willingness to adopt in multiple models (standardised βs range from −0.25 to −0.60, *p* < 0.05), while educational attainment was positively associated with willingness to adopt for several actions (standardised βs range from 0.25 to 0.60, *p* < 0.05). Involvement in food cooking was negatively associated with willingness to adopt for a subset of actions (standardised βs range from −0.20 to −0.50, *p* < 0.05). Among cognitive variables, perceived importance was the most consistent predictor of willingness to adopt, reaching statistical significance for 15 of 18 actions with large effect sizes (smallest β = 0.60, *p* < 0.001). Knowledge was significantly associated with willingness to adopt for a smaller number of actions, with both positive and negative coefficients observed across models. Perceived value of undertaking the action was positively associated with willingness to adopt for several actions (standardised βs range from 0.20 to 0.50, *p* < 0.05), while willingness to make small changes was negatively associated with willingness to adopt in a limited number of models. Perceived impact was statistically significant for a limited number of actions (6 models), with modest to moderate effect sizes (standardised βs range from 0.25 to 0.55, *p* < 0.05).

## 4. Discussion

A number of key findings emerge from this study. First, our findings show that knowledge was unevenly distributed across our healthy sustainable actions. Participants exhibited greater knowledge of actions that aligned with general health recommendations, such as drinking the recommended amount of water per day, and snacking on fruits, vegetables, and nuts. However, knowledge gaps were evident for less common but environmentally impactful actions, including replacing meat with pulses and consuming offal. This may suggest that public understanding of healthy sustainable diets is largely confined to behaviours that align with general health advice, with less recognition of environmental impacts. Previous research suggests that consumers often overlook the interconnection between health and sustainability and prioritise health-related aspects over environmental concerns [36,37,38,39] while recognising that most public health campaigns focus primarily on health outcomes, rather than addressing both health and environmental benefits [38]. These findings highlight the importance of clearly communicating the environmental parameters relevant to healthy sustainable diets, such as greenhouse gas emissions, land use, water use, and biodiversity, to enable individuals to understand why certain actions are considered sustainable.

To support interpretation of these patterns, actions were considered in relation to their dominant behavioural characteristics, including (i) habitual practices (e.g., routine food management behaviours), (ii) health-normative substitutions (e.g., swaps aligned with existing dietary advice), and (iii) norm-challenging dietary substitutions (e.g., actions involving unfamiliar foods or reductions in meat consumption). These groupings were not imposed analytically and were not used in statistical modelling but were applied conceptually to aid the interpretation of broader trends observed across knowledge, perceived importance, current uptake, and willingness to adopt.

It is important to note that knowledge in our study was operationalised as recognition of actions commonly promoted as healthy and sustainable, reflecting applied, everyday understanding rather than detailed scientific knowledge. As such, recognition-based measures may overestimate depth of understanding, particularly for familiar actions, and should be interpreted as indicative of awareness rather than comprehensive knowledge. This pattern is consistent with prior work suggesting that individuals may overestimate their understanding of familiar health-related behaviours, a phenomenon often discussed in relation to the Dunning–Kruger effect, whereby perceived knowledge exceeds objective understanding [40,41].

Independent of the effects in knowledge, participants perceived some healthy sustainable actions as more important than others. Actions such as using the freezer more, drinking the recommended amount of water per day, avoiding leftovers, and having two days without meat consumption were rated as highly important. In contrast, actions like consuming offal or replacing meat with future foods were perceived as less important. Perceived importance was generally higher for actions that were more familiar or already embedded in common dietary advice, while less conventional or culturally unfamiliar actions were viewed as less important. Previous research has shown that individuals are more likely to value and adopt dietary behaviours perceived as congruent with existing habits, health messages, or social norms [27,38]. Conversely, novel, culturally incongruent or currently unfamiliar foods, such as offal or alternative proteins, often evoke lower acceptance due to food neophobia, perceived disgust, or a lack of perceived value [27,36,38]. Specific to healthy sustainable eating, some research also highlights a failure in consumers to recognise the links between diet and sustainability, or the extent of the contribution of dietary choices to sustainability goals [9,13,39,42]. For actions that currently hold lower perceived importance, value may be gained from emphasising the broader benefits of these actions, including elements of sustainability.

Our findings showed similar variability in current engagement with sustainable actions. Greater engagement was observed for actions that were convenient and routine-based, such as using the freezer more and purchasing locally produced foods. In contrast, actions requiring greater dietary change or unfamiliar food choices, such as replacing meat with pulses or consuming fish lower in the food chain, were less commonly adopted. These findings are consistent with previous research showing that individuals are more likely to adopt behaviours that are habitual and accessible [23,42,43]. However, while the most widely known actions tended to reflect established health advice, we found actual engagement was more strongly predicted by knowledge and the perceived importance of healthy sustainable eating, including environmental concern. Associations between engaging in healthy sustainable eating, knowledge of and its perceived importance have been found previously [8,14]. These findings may suggest a potential disconnect: individuals may be more aware of potential health-promoting actions of healthy sustainable eating but are more likely to engage with them when they both understand and value their broader significance, including the potential sustainability benefits. These findings highlight the need for public health strategies to better communicate the environmental benefits of dietary actions [7,13,39,42], potentially enhancing engagement by strengthening their perceived importance for sustainability.

These factors were also more influential than most demographic variables, highlighting the central role of knowledge and perceived importance in shaping sustainable dietary action. However, demographic, lifestyle, and some other cognitive factors also shaped current engagement with sustainable dietary behaviours to some degree. Age, education, and frequency of cooking notably influenced several sustainable actions, such that greater current uptake was reported by younger and more educated individuals, who cooked more often. These effects have been previously reported [8,14]. Given the exploratory and comparative nature of the analyses, emphasis is placed on consistent patterns of association across multiple actions rather than isolated statistically significant effects.

Despite varying levels of current engagement, our results showed a strong willingness to adopt certain sustainable actions. Willingness was highest for actions that already aligned with prevailing social norms in the UK, such as avoiding leftovers, and was lower for actions that required unfamiliar behaviour or challenged established cultural norms [12,38]. Furthermore, several sustainable actions emerged as underperformed yet they were accompanied by an expressed willingness to adopt. These included using the freezer more, having two meat-free days per week, swapping white for wholegrain foods, swapping ruminant meat for chicken, and only snacking on fruits, vegetables, and nuts. These actions may represent important areas for behavioural intervention where readiness to change may already be present. Importantly, identifying actions that were both underperformed and accompanied by willingness to adopt would not have been possible through willingness ratings alone, as willingness does not distinguish between actions already embedded in routine behaviour and those representing genuine opportunity for change. Highlighting these underperformed actions that people are willing to perform may, therefore, support the development of targeted interventions that prioritise changes with the greatest potential for adoption.

Among the cognitive factors investigated, perceived importance emerged as the most consistent and strongest predictor, significantly influencing willingness across nearly all actions examined. While previous research has identified perceived importance as a relevant factor in healthy sustainable behaviours [12,38], our findings extend this by showing its broader predictive strength relative to other factors, across a wide range of healthy sustainable dietary actions. Our findings indicate that perceived importance may play a key role in bridging an intention–behaviour gap and may represent a valuable focus for the development of behavioural interventions. Our findings align with the Health Belief Model [16], which posits that individuals are more likely to undertake health-related actions when they perceive them as beneficial and personally meaningful. More broadly, these findings align with value-based and motivation-focused models of behaviour change, which emphasise the central role of perceived relevance and benefit over information provision alone in shaping action [15,16,17]. To promote dietary change at scale, public health strategies may need to go beyond awareness-raising in order to enhance the subjective value individuals assign to healthy sustainable actions. Interventions that highlight personal benefits (e.g., improved health, affordability), alongside broader collective outcomes (e.g., environmental sustainability, food system resilience), may be particularly effective in translating intention into action.

Perceived value, conceptualised in our study as the extent to which an action is seen as worthwhile or non-futile even if only I do it, emerged as a significant positive predictor for several actions. Notably, higher perceived value was associated with greater willingness to adopt future foods, ensure all meat is farmed in the UK, consume fruits and vegetables with ethical trade stamps, and consume locally grown fruits and vegetables. These findings suggest that when individuals view their own dietary actions as effective, they are more likely to consider adopting them. This supports earlier research identifying perceived value as a key barrier to engagement in pro-environmental behaviours [5]. Conversely, when actions are perceived as ineffective or inconsequential, even those with clear sustainability benefits may be disregarded. This highlights the importance of directly addressing perceptions of futility in public health messaging. Framing sustainable eating as rewarding through collective impact may enhance its perceived value. Emphasising the contribution of individual actions to broader social and environmental goals may help to counter perceptions of futility.

For perceptions of small changes, the negative relationships found suggest that the belief that minor adjustments are sufficient to contribute to sustainability may, in some instances, reduce willingness to engage with more demanding or unfamiliar actions, such as replacing meat with future foods or choosing Fairtrade products, or alternatively, that these actions are not seen as ‘small’. While public health messaging often frames small changes as a low-barrier strategy to facilitate engagement, and familiarity with broader sustainability concepts may support understanding of the relevance of such actions [16], such framing must align with how these actions are perceived. This is consistent with previous research questioning the general effectiveness of ‘small change’ strategies, particularly when the target behaviour is novel or unfamiliar [44].

Among the cognitive factors, knowledge and impact were only weakly associated with willingness to undertake actions that were not currently done. These findings confirm that knowledge of and recognition of an action’s broader environmental value does not necessarily translate into behavioural intention. The inconsistent associations observed for knowledge may also reflect greater awareness of practical and psychological barriers among more knowledgeable individuals. Those with higher awareness of sustainable dietary recommendations may simultaneously hold more accurate perceptions of the effort, cost, inconvenience, or social challenges associated with implementing these actions, which could temper willingness to adopt them [5,23]. This interpretation aligns with research suggesting that increased awareness does not necessarily increase motivation and may, in some cases, heighten perceived difficulty or reduce the perceived feasibility of behaviour change [43]. Where perceived impact was significant, effects were limited to a small number of actions and were generally weaker than those observed for perceived importance and perceived value. Similarly, the weak or negative associations observed for ‘small change’ framing may indicate that presenting sustainability as incremental or low-effort can reduce perceived urgency or moral salience for more demanding actions. Previous research suggests that interventions framed as minimal-impact behaviours may inadvertently signal limited effectiveness, thereby weakening motivation to engage in norm-challenging dietary transitions [22,24]. These findings highlight the importance of distinguishing between knowledge, perceived impact, perceived importance, and value, and suggest that enhancing the perceived importance and value of impactful dietary actions may be necessary to support their wider adoption. Taken together, our findings suggest that both perceived importance and perceived value appear to be central to motivation, although in distinct ways: importance relates to personal relevance, while value relates to perceived effectiveness.

Building on these findings, interventions aiming to promote healthy sustainable actions may benefit from explicitly targeting perceived importance and perceived value through concrete framing strategies. For example, actions that were unfamiliar or perceived as low in importance, such as consuming offal or replacing meat with alternative proteins, could be reframed in terms of affordability, culinary tradition, or cultural identity, rather than solely environmental benefit, as familiarity and identity congruence have been shown to influence acceptance of sustainable foods [27,38]. Emphasising cost savings, links to traditional cuisines, or alignment with familiar meals may increase the perceived personal relevance of such actions. Similarly, perceived value may be strengthened by directly countering perceptions of futility. Interventions could highlight collective impact by communicating how small individual dietary changes, when adopted across populations, contribute meaningfully to environmental outcomes, which has been shown to enhance motivation and perceived effectiveness [45]. Providing population-level equivalence messages (e.g., aggregated reductions in emissions or resource use) may help individuals view their own actions as worthwhile, even when undertaken in isolation. Together, these strategies illustrate how insights into perceived importance and perceived value can be translated into practical intervention approaches without relying on information provision alone [46].

Willingness to adopt sustainable actions that were not currently undertaken was also shaped by similar demographic and lifestyle factors to those influencing current engagement. Age and education were again strong demographic predictors, as was food cooking, although food cooking was negatively associated with willingness to undertake actions that were not currently undertaken. These findings may suggest resistance if actions are perceived as effortful or require cooking, if actions are perceived as unlikely to be acceptable to other family members or those for whom the cooking is undertaken, or may suggest that individuals are more positive towards undertaking healthy sustainable actions if they do less cooking. Resistance to healthy sustainable food consumption as a result of the effort involved in cooking, low confidence in cooking abilities, and likely wasted effort due to low acceptance by other diners have previously been reported [8,13,14,39,47,48].

A notable strength of our study is the focus on specific, actionable dietary changes, rather than broad or abstract concepts of healthy sustainable eating. This targeted approach allows for the identification of practical intervention opportunities—actions that participants are not currently engaging in but are willing to adopt. By systematically comparing knowledge, perceived importance, current engagement, and willingness across a wide range of specific dietary actions within the same sample, this study extends prior work that has largely focused on single behaviours or broad dietary patterns [10,49,50]. The highly ranked actions, such as ‘using the freezer more’, ‘having two days a week with no meat’, and ‘swapping ruminant beef for chicken’, represent realistic and impactful targets for dietary change. Our study differs from previous research by integrating knowledge, perceived importance, and perceived value, and examining their influence on individuals’ willingness to adopt healthy sustainable dietary actions. Perceived importance emerged as one of the more influential cognitive factors across actions, particularly in comparison to knowledge and impact. Our results suggest that interventions designed to promote healthy sustainable dietary practices should enhance individuals’ perceptions of the personal importance and value of specific actions. Limited effect sizes in the associations with the demographic and lifestyle variables also suggest that interventions focused on perceived importance and perceived value are likely to be effective for a wide-ranging audience, without the necessity for highly segmented strategies. However, there may be additional benefits to targeting specific subgroups, such as older adults and individuals with lower educational attainment.

Despite our effort to keep the questionnaire as short as possible, it took an average of eighteen minutes to complete. Although the pilot participants did not complain and completed it in a timely manner, this may have affected the overall response rate. Concerns over questionnaire length limited particularly the additional factors that were investigated in relation to healthy sustainable dietary behaviour. It is important to consider that food environments strongly influence what people can feasibly choose. Availability, affordability, and the visibility of healthy sustainable foods can either support or hinder engagement, suggesting that environmental and structural changes may be necessary alongside individual-level education. Other cognitive factors, e.g., self-efficacy, confidence, or perceived behavioural control [23,43,44,48], may also play a role. Other forms of response bias might also include a lower response from people who do not like the notion of adopting sustainable eating practices. Although our measure of current engagement was validated (see Section 2.3), it is also important to note potential limitations of recall bias, as participants may have difficulty accurately recalling their food consumption, leading to potential over- or under-reporting of food intake due to social desirability bias [28], especially in relation to sustainable behaviours. Research shows that individuals may over-report their intake of sustainable foods because it aligns with perceived societal norms. We must also consider that our sample was over-represented by females and based in the UK; thus, our findings may not be generalisable to males or other countries with different food systems, sustainability practices, or cultural norms around food. Given the observed overrepresentation of females, it is possible that mean levels of perceived importance, willingness, and self-reported uptake may be overestimated if women in the UK are more likely to endorse health-and sustainability-oriented attitudes or to report socially desirable behaviours. This sampling imbalance should, therefore, be considered when interpreting the magnitude and generalizability of the observed effects. Additionally, although we examined constructs such as knowledge, perceived importance, current engagement, and willingness as separate constructs for analytical clarity, these elements are inherently interconnected and likely influence one another dynamically in real-world decision-making. Given the large number of statistical tests conducted across multiple actions and models, there is an increased risk of Type I error. As the primary aim of the analyses was comparative and exploratory, formal correction for multiple comparisons was not applied. The findings are, therefore, interpreted conservatively, with emphasis placed on consistent patterns across actions rather than isolated statistically significant effects. Lastly, because of the cross-sectional nature of the study, the direction of causality is unknown.

## 5. Conclusions

Our research provides novel insights into the psychological and demographic drivers of healthy sustainable dietary behaviours by examining specific actions in relation to knowledge, current uptake, and willingness to adopt. While knowledge and current engagement were highest for familiar, health-aligned behaviours, actions with stronger environmental benefits were less well understood and less often adopted. When considering willingness to undertake actions that were not currently practised, perceived importance and perceived value emerged as influential positive predictors of willingness to act, highlighting the need for public health strategies that go beyond awareness-raising to emphasise the significance and value of dietary actions. These cognitive factors also showed broad applicability across demographic and lifestyle variables, suggesting the potential for widely effective interventions.

## Figures and Tables

**Table 1:** Participant characteristics (N = 635).

Characteristic	Value *
**Age**	
18–24 years old	129 (20.3%)
25–34 years old	97 (15.2%)
35–44 years old	119 (18.7%)
45–54 years old	104 (16.3%)
55–64 years old	95 (14.9%)
65+ years old	91 (14.3%)
**Gender**	
Male	205 (32.8%)
Female	430 (67.5%)
**Education level**	
No qualifications	9 (1.4%)
GCSEs, Post 16, or equivalent	44 (6.9%)
A-levels, Post 18, or equivalent	234 (36.8%)
Foundation degree or equivalent	59 (9.2%)
Bachelor’s degree or equivalent	139 (21.8%)
Master’s degree or equivalent	72 (11.3%)
Professional degree or equivalent	59 (9.2%)
Doctorate degree	19 (2.9%)
**Employment status**	
Full-time employed	206 (32.4%)
Part-time employed	136 (21.4%)
Self-employed	49 (7.7%)
Unemployed	22 (3.3%)
Student	98 (15.4%)
Retired	106 (16.6%)
Unable to work	18 (2.8%)

* The values provided are the number of responses and percentage of all responses in the specific category.

**Table 2:** Integrated summary of knowledge, perceived importance, current uptake, willingness to adopt, and NCU-W scores across healthy sustainable dietary actions (N = 635). Colour intensity reflects rank groupings derived from paired-samples comparisons, with darker shading indicating higher-ranked actions and lighter shading indicating lower-ranked actions within each construct. Reported t-values represent one-sample tests against the neutral midpoint (for perceived importance, willingness, and NCU-W), indicating whether mean values differ significantly from a neutral midpoint.

	Knowledge	Perceived Importance	Current Uptake	Willingness to Adopt	NCU-W Scores
Healthy Sustainable Action	Number of Respondents	Mean (Std. Dev) *p* (t)	Average Times per Week (Std. Dev)	Mean (Std. Dev) *p* (t)	Mean (Std. Dev) *p* (t)
Drink the recommended amount of water per day	459 (72%)	1.09 (1.14) <0.001 (24.04)	4.29 (2.59)	1.37 (0.95) <0.001 (36.08)	3.65 (2.35) <0.001 (50.66)
Ensure you have no leftovers	368 (57%)	0.92 (1.17) <0.001 (19.87)	4.11 (2.67)	1.34 (0.99) <0.001 (33.68)	3.29 (2.72) <0.001 (30.46)
Use your freezer more: If you can’t eat it before it perishes, freeze it.	600 (94%)	1.49 (0.81) <0.001 (46.15)	4.00 (2.71)	1.58 (0.81) <0.001 (48.39)	4.20 (2.09) <0.001 (50.66)
Only snack on fruits, vegetables, or nuts.	497 (78%)	0.58 (1.02) 0.03 (14.41)	2.13 (2.27)	0.93 (1.15) <0.001 (20.19)	1.95 (2.58) <0.001 (19.02)
Swap white foods for wholegrain foods.	365 (57%)	0.64 (1.13) <0.001 (14.44)	2.17 (2.43)	1.01 (1.17) <0.001 (21.32)	1.92 (2.86) <0.001 (16.95)
Ensure all meat bought is from animals farmed in the UK.	411 (64%)	0.97 (1.06) <0.001 (23.11)	2.04 (2.57)	0.87 (1.19) <0.001 (17.36)	1.82 (2.98) <0.001 (15.37)
Swap your normal tea and coffee brand for a Fairtrade substitute.	350 (55%)	0.44 (1.14) <0.001 (9.78)	1.97 (2.86)	0.40 (1.43) <0.001 (6.93)	0.54 (3.45) <0.001 (3.97)
Have two days a week with no meat consumption.	507 (79%)	1.04 (1.08) <0.001 (24.25)	1.86 (2.49)	0.89 (1.30) <0.001 (16.52)	1.90 (2.91) <0.001 (16.48)
Ensure all vegetables in your diet are seasonal.	448 (70%)	0.97 (1.01) <0.001 (24.24)	1.80 (2.24)	0.83 (1.11) <0.001 (18.84)	1.98 (2.74) <0.001 (18.21)
Aim to eat the whole vegetable when preparing meals	361 (56%)	0.62 (1.10) <0.001 (14.23)	1.74 (2.39)	0.49 (1.30) <0.001 (9.54)	1.07 (3.29) <0.001 (8.23)
Only eat fruits and vegetables that have Fairtrade or ethical trade certification stamps.	419 (65%)	0.60 (1.10) <0.001 (13.75)	1.30 (2.02)	0.40 (1.28) <0.001 (7.91)	0.78 (3.09) <0.001 (6.35)
Swap any beef, lamb, or pork intake for chicken.	207 (32%)	0.17 (1.07) <0.001 (4.17)	1.24 (1.88)	0.88 (1.21) <0.001 (17.02)	0.72 (2.86) <0.001 (6.39)
Swap all meat intake for pulses	253 (39%)	0.23 (1.27) <0.001 (4.61)	1.21 (2.27)	0.11 (1.47) 0.06 (1.88)	−0.24 (3.45) 0.07 (−1.79)
Ensure all fruit and veg are bought from local farm shops or local market equivalents.	409 (64%)	0.74 (0.99) <0.001 (19.02)	1.07 (1.95)	0.24 (1.28) <0.001 (4.84)	0.67 (2.86) <0.001 (5.96)
Choose to eat organic fruits and vegetables versus their non-organic counterparts.	277 (43%)	0.45 (1.10) <0.001 (10.31)	0.93 (1.78)	0.23 (1.29) <0.001 (4.48)	0.20 (3.06) 0.08 (1.70)
Eat more fish that are low in the food chain	195 (30%)	0.12 (−1.07) <0.001 (−3.54)	0.46 (1.18)	0.03 (1.41) 0.55 (0.50)	−0.59 (2.84) <0.001 (−5.28)
Swap one portion of meat intake for a ‘future food’ substitute	224 (25%)	−0.17 (1.22) <0.001 (−3.54)	0.18 (0.91)	−0.91 (1.26) <0.001 (−17.10)	−2.61 (2.50) <0.001 (−26.32)
Swap any beef, lamb, or pork intake for offal	42 (6%)	−0.55 (1.03) <0.001 (−13.59)	0.14 (0.73)	−0.95 (1.27) <0.001 (−17.48)	−2.86 (2.36) <0.001 (−30.53)

## Data Availability

The data supporting the findings of this study are available from the corresponding author upon reasonable request. Public access is restricted whilst related work is ongoing.

## Supplementary Materials

The following supporting information can be downloaded at https://www.mdpi.com/article/10.3390/nu18030534/s1: Table S1: Participants’ current frequency of certain food items assuming a standard frequency throughout time; Table S2: Means, standard deviations, and correlation coefficients of what people are currently doing versus their actual intakes; Table S3: Results of all regression analyses for the sustainable actions the UK population currently do; Table S4: Results of all regression analyses for the sustainable actions the UK population sample are not currently doing but are willing to adopt (the composite NCU-W score).

## Abbreviations

The following abbreviations are used in this manuscript:

FFQ	Food Frequency Questionnaire
UK	United Kingdom
NCU-W	Not Currently Undertaking but Willing (Composite Score)
