# Supplementary material for: Knowledge, Perceived Importance, Current Uptake, and Willingness to Adopt Healthy Sustainable Dietary Actions: A Cross-Sectional Study of UK Adults
Source: Nutrients. 2026 Feb 5;18(3):534. doi: 10.3390/nu18030534 (PMC12899006; doi:10.3390/nu18030534)
Supplement: Supplementary file 1 [file nutrients-18-00534-s001.zip › nutrients-4101267-supplementary.pdf]

Knowledge, Perceived Importance, Current Uptake, and Willingness to Adopt Healthy Sustainable Dietary Actions: A Cross-Sectional Study of UK Adults

Danielle J Guy, Jeffery Bray and Katherine M Appleton

SUPPLEMENTARY MATERIALS

Copy of questionnaire:

1. **How often do you eat each of the following dairy products?**
Please place a tick in the appropriate box.

	Every day	3-5 times a week	1-2 times a week	1-2 times a fortnight	1-2 times a month	Less than once a month	Never
Milk (including in tea/coffee)	☐	☐	☐	☐	☐	☐	☐
Butter	☐	☐	☐	☐	☐	☐	☐
Margarine (e.g., stork, clover)	☐	☐	☐	☐	☐	☐	☐
Low fat spreads (e.g., Lurpak spreadable)	☐	☐	☐	☐	☐	☐	☐
Yoghurts	☐	☐	☐	☐	☐	☐	☐
Creams (e.g., whipped)	☐	☐	☐	☐	☐	☐	☐
Eggs – fried	☐	☐	☐	☐	☐	☐	☐
Eggs – not fried (boiled, poached, in baking)	☐	☐	☐	☐	☐	☐	☐
Cheese and/or egg dishes (e.g., pizza, quiche)	☐	☐	☐	☐	☐	☐	☐
Soy, rice, almond, or coconut milk (dairy alternatives)	☐	☐	☐	☐	☐	☐	☐
Non-dairy cheeses	☐	☐	☐	☐	☐	☐	☐
Non-dairy margarine	☐	☐	☐	☐	☐	☐	☐
Other non-dairy alternatives	☐	☐	☐	☐	☐	☐	☐

2. How often do you eat each of the following protein products?

Please place a tick in the appropriate box.

	Every day	3-5 times a week	1-2 times a week	1-2 times a fortnight	1-2 times a month	Less than once a month	Never
Beef (roast/steak/mince)	☐	☐	☐	☐	☐	☐	☐
Lamb (roast/steak/mince)	☐	☐	☐	☐	☐	☐	☐
Pork (roast/steak/mince)	☐	☐	☐	☐	☐	☐	☐
Chicken, Turkey, or other poultry	☐	☐	☐	☐	☐	☐	☐
Bacon or Gammon	☐	☐	☐	☐	☐	☐	☐
Processed meat (e.g., ham, corned beef)	☐	☐	☐	☐	☐	☐	☐
Meat pies, sausage rolls, or pasties	☐	☐	☐	☐	☐	☐	☐
Sausages or beefburgers	☐	☐	☐	☐	☐	☐	☐
Liver, Kidney, Pâté (any other offal products)	☐	☐	☐	☐	☐	☐	☐
Fish and Seafood (not fried)	☐	☐	☐	☐	☐	☐	☐
Fish and Seafood (fried)	☐	☐	☐	☐	☐	☐	☐
Fish and Seafood (canned e.g., tuna)	☐	☐	☐	☐	☐	☐	☐
Soy products (e.g., tofu, tempeh, edamame)	☐	☐	☐	☐	☐	☐	☐
Lentils	☐	☐	☐	☐	☐	☐	☐
Chickpeas	☐	☐	☐	☐	☐	☐	☐
Nuts	☐	☐	☐	☐	☐	☐	☐
'Future Foods' (e.g., mussels, algae, insects)	☐	☐	☐	☐	☐	☐	☐
Protein shakes or equivalent	☐	☐	☐	☐	☐	☐	☐

3. How often do you eat each of the following bread products?
Please place a tick in the appropriate box.

	Every day	3-5 times a week	1-2 times a week	1-2 times a fortnight	1-2 times a month	Less than once a month	Never
White bread	☐	☐	☐	☐	☐	☐	☐
Brown/Granary bread	☐	☐	☐	☐	☐	☐	☐
Wholemeal bread	☐	☐	☐	☐	☐	☐	☐
Other bread items (e.g., naan, pitta, tortilla wraps)	☐	☐	☐	☐	☐	☐	☐

4. How often do you eat each of the following fruits and vegetables?
Please place a tick in the appropriate box.

	Every day	3-5 times a week	1-2 times a week	1-2 times a fortnight	1-2 times a month	Less than once a month	Never
Apples, Pears	☐	☐	☐	☐	☐	☐	☐
Oranges, Grapefruits, or other citrus fruits	☐	☐	☐	☐	☐	☐	☐
Bananas	☐	☐	☐	☐	☐	☐	☐
Grapes	☐	☐	☐	☐	☐	☐	☐
Berries (e.g., strawberries, raspberries, blueberries)	☐	☐	☐	☐	☐	☐	☐
Melons (e.g., watermelon, honeydew)	☐	☐	☐	☐	☐	☐	☐
Green vegetables (e.g., peas, broccoli)	☐	☐	☐	☐	☐	☐	☐
Carrots, Tomatoes (fresh or canned)	☐	☐	☐	☐	☐	☐	☐
Other vegetables (including salad vegetables)	☐	☐	☐	☐	☐	☐	☐
Baked Beans	☐	☐	☐	☐	☐	☐	☐
Other beans / Lentils (e.g., Dahl)	☐	☐	☐	☐	☐	☐	☐
Vegetable dishes (e.g., stew, curry – NO Meat)	☐	☐	☐	☐	☐	☐	☐
Potatoes (chips, roasted, fried)	☐	☐	☐	☐	☐	☐	☐
Potatoes (not fried, boiled, baked)	☐	☐	☐	☐	☐	☐	☐

5. What do you think a 'healthy sustainable diet' consists of?

Please tick all the answers that you think are sustainable actions which contribute to having a healthy sustainable diet.

☐	Swap all meat intake for pulses (beans, lentils, etc).
☐	Ensure all vegetables in your diet are seasonal.
☐	Ensure all meat bought is from animals farmed in the UK.
☐	Eat more beef than chicken.
☐	Ensure you have no leftovers.
☐	Eat the whole vegetables when preparing meals (root greens, stalks, peelings, etc).
☐	Eat more tropical fruits (e.g., avocadoes, papayas).
☐	Swap one portion of meat intake for a 'future food' substitute (e.g., crickets, seaweed).
☐	Drink the recommended amount of water for the day (approximately 1.5-2 litres).
☐	Swap white foods for wholegrain foods.
☐	Eat organic fruits and vegetables versus their non-organic counterparts.
☐	Only eat non-free-range eggs.
☐	Swap your normal tea or coffee brand for a Fair-trade substitute.
☐	Eat fish that are low in the food chain (e.g., sardines, anchovies, pilchards).
☐	Swap any beef, lamb, or pork intake for chicken.
☐	Ensure all fruit and vegetables are bought from local farm shops or local market equivalents.
☐	Snack on fruits, vegetables, or nuts.
☐	Only consume fish that are high in the food chain (e.g., salmon, trout).
☐	Eat fruits and vegetables that have Fair-trade or ethical trade certification stamps.
☐	Use your freezer more: If you can't eat it before it perishes, freeze it.
☐	Have two days a week with no meat consumption.
☐	Discard food when it has reached its best-before date even if it is still safe to consume.
☐	Swap any beef, lamb, or pork intake for offal (e.g., kidneys, liver, black pudding).

6. What do you think is the most important in achieving a healthy sustainable diet?

Please read each sustainable action and tick the option which indicates how important you believe this sustainable action is in achieving a healthy sustainable diet.

	Very important	Somewhat important	Neutral	Somewhat unimportant	Not at all important
Swap all meat intake for pulses (e.g., lentils, beans)	☐	☐	☐	☐	☐
Ensure all vegetables in your diet are seasonal	☐	☐	☐	☐	☐
Ensure all meat bought is from animals farmed in the UK	☐	☐	☐	☐	☐
Ensure you have no leftovers	☐	☐	☐	☐	☐
Eat the whole vegetable when preparing meals (root greens, stalks, and peelings)	☐	☐	☐	☐	☐
Swap portions of meat for ‘future food’ substitutes (e.g., crickets, seaweed)	☐	☐	☐	☐	☐
Drink the recommended amount of water per day (approx. 1.5-2 litres)	☐	☐	☐	☐	☐
Eat only wholegrain foods (no white foods)	☐	☐	☐	☐	☐
Only eat organic fruits and vegetables	☐	☐	☐	☐	☐
Drink Fair-trade tea or coffee	☐	☐	☐	☐	☐
Eat fish that are low in the food chain (e.g., mackerel, sardines)	☐	☐	☐	☐	☐
Swap any beef, lamb, or pork intake for chicken	☐	☐	☐	☐	☐
Buy all fruit and vegetables from local farm shops or local market equivalents	☐	☐	☐	☐	☐
Use your freezer more: If you can’t it before it perishes, freeze it	☐	☐	☐	☐	☐

7. Do you currently do any of these actions to make your diet more sustainable?

Please read each sustainable action and select the option which indicates how often you carry out this sustainable action.

	Every day	3-5 times a week	1-2 times a week	1-2 times a fortnight	1-2 times a month	Less than once a month	Never
Swap all meat intake for pulses (e.g., lentils, beans)	☐	☐	☐	☐	☐	☐	☐
Ensure all vegetables in your diet are seasonal	☐	☐	☐	☐	☐	☐	☐
Ensure all meat bought is from animals farmed in the UK	☐	☐	☐	☐	☐	☐	☐
Ensure you have no leftovers	☐	☐	☐	☐	☐	☐	☐
Eat the whole vegetable when preparing meals (root greens, stalks, and peelings)	☐	☐	☐	☐	☐	☐	☐
Swap portions of meat for ‘future food’ substitutes (e.g., crickets, seaweed)	☐	☐	☐	☐	☐	☐	☐
Drink the recommended amount of water per day (approx. 1.5-2 litres)	☐	☐	☐	☐	☐	☐	☐
Eat only wholegrain foods (no white foods)	☐	☐	☐	☐	☐	☐	☐
Only eat organic fruits and vegetables	☐	☐	☐	☐	☐	☐	☐
Drink Fair-trade tea or coffee	☐	☐	☐	☐	☐	☐	☐
Eat fish that are low in the food chain (e.g., mackerel, sardines)	☐	☐	☐	☐	☐	☐	☐
Swap any beef, lamb, or pork intake for chicken	☐	☐	☐	☐	☐	☐	☐
Buy all fruit and vegetables from local farm shops or local market equivalents	☐	☐	☐	☐	☐	☐	☐
Use your freezer more: If you can’t it before it perishes, freeze it	☐	☐	☐	☐	☐	☐	☐
Only snack on fruits, vegetables, and nuts	☐	☐	☐	☐	☐	☐	☐
Have no meat consumption	☐	☐	☐	☐	☐	☐	☐
Eat offal (e.g., kidneys, liver)	☐	☐	☐	☐	☐	☐	☐

8. Are you willing to do any of these to make your diet more sustainable?

Please read each of sustainable action and select the option which indicates how willing you would be to carry out this sustainable action going forward. If there are some questions that do not apply to you (e.g., you are vegan or vegetarian) please select N/A.

	Very likely	Unlikely	Neutral	Likely	Very likely	N/A
For at least two days a week, swap all meat intake for pulses (e.g., lentils, beans)	☐	☐	☐	☐	☐	☐
For at least two days a week, ensure all vegetables in your diet are seasonal	☐	☐	☐	☐	☐	☐
For at least two days a week, ensure all meat bought is from animals farmed in the UK	☐	☐	☐	☐	☐	☐
For at least three days a week, ensure you have no leftovers	☐	☐	☐	☐	☐	☐
Aim to eat the whole vegetable when preparing meals (root greens, stalks, and peelings)	☐	☐	☐	☐	☐	☐
For at least two days a week, swap one portion of meat intake for 'future food' substitutes (e.g., crickets, seaweed)	☐	☐	☐	☐	☐	☐
Ensure that you drink the recommended amount of water per day (approx. 1.5-2 litres)	☐	☐	☐	☐	☐	☐
For at least two days a week, swap white foods for wholegrain foods	☐	☐	☐	☐	☐	☐
For at least two meals a week, choose to eat organic fruits and vegetables versus their non-organic counterparts	☐	☐	☐	☐	☐	☐
For at least one week, swap your normal tea or coffee brand for a Fair-trade substitute	☐	☐	☐	☐	☐	☐
Eat more fish that are low in the food chain (e.g., sardines)	☐	☐	☐	☐	☐	☐
For at least two days a week, swap any beef, lamb, or pork intake for chicken	☐	☐	☐	☐	☐	☐

For at least two days a week, ensure all fruit and vegetables bought from local farm ships or local market equivalent	☐	☐	☐	☐	☐	☐
Use your freezer more: If you can’t it before it perishes, freeze it	☐	☐	☐	☐	☐	☐
For at least two days a week, only snack on fruits, vegetables, and nuts	☐	☐	☐	☐	☐	☐
For at least two days a week with no meat consumption	☐	☐	☐	☐	☐	☐
For at least two days a week, swap any beef, lamb, or pork intake for offal (e.g., kidneys, liver)	☐	☐	☐	☐	☐	☐

9. Please list any **barriers** which currently <u>prevent you</u> from adopting a more sustainable diet:

10. Please list anything that **currently helps you** to adopt a more sustainable diet:

11. I think sustainability is important.

☐ Strongly agree

☐ Agree

☐ Neither agree nor disagree

☐ Disagree

☐ Strongly disagree

12. I think making a big change to make my diet more sustainable would be unachievable.

☐ Strongly agree

☐ Agree

☐ Neither agree nor disagree

☐ Disagree

☐ Strongly disagree

13. I think it is important to live a lifestyle that is sustainable.

☐ Strongly agree

☐ Agree

☐ Neither agree nor disagree

☐ Disagree

☐ Strongly disagree

14. My dietary actions alone can make a difference.

☐ Strongly agree

☐ Agree

☐ Neither agree nor disagree

☐ Disagree

☐ Strongly disagree

15. I care about the environment.

☐ Strongly agree

☐ Agree

☐ Neither agree nor disagree

☐ Disagree

☐ Strongly disagree

16. I am willing to make a small change to make my diet more sustainable.

☐ Strongly agree

☐ Agree

☐ Neither agree nor disagree

☐ Disagree

☐ Strongly disagree

17. I think it is important to live a lifestyle that is environmentally friendly.

☐ Strongly agree

☐ Agree

☐ Neither agree nor disagree

☐ Disagree

☐ Strongly disagree

18. Everyone needs to eat more sustainably for this to make a difference.

☐ Strongly agree

☐ Agree

☐ Neither agree nor disagree

☐ Disagree

☐ Strongly disagree

19. I know what a healthy diet consists of.

☐ Strongly agree

☐ Agree

☐ Neither agree nor disagree

☐ Disagree

☐ Strongly disagree

20. I know what a sustainable diet consists of.

☐ Strongly agree

☐ Agree

☐ Neither agree nor disagree

☐ Disagree

☐ Strongly disagree

21. I understand the impact my diet has on my health.

☐ Strongly agree

☐ Agree

☐ Neither agree nor disagree

☐ Disagree

☐ Strongly disagree

22. I understand the impact my diet has on the environment.

☐ Strongly agree

☐ Agree

☐ Neither agree nor disagree

☐ Disagree

☐ Strongly disagree

Demographic Information:

9. What is your age?

☐ 18-24 years

☐ 25-34 years

☐ 35-44 years

☐ 45-54 years

☐ 55-64 years

☐ 65+ years

10. What gender do you identify as?

☐ Male

☐ Female

☐ Non-binary / third gender

☐ Other

☐ Prefer not to say

11. What is the highest level of education you have completed?

☐ No qualifications

☐ GCSEs or equivalent

☐ A-levels or equivalent

☐ Foundation degree or equivalent

☐ Bachelor’s degree or equivalent

☐ Master’s degree or equivalent

☐ Professional degree

☐ Doctorate degree

12. What is your current employment status?

☐ Full-time employed

☐ Part-time employed

☐ Self-employed

☐ Unemployed (looking for work)

☐ Unemployed (not looking for work)

☐ Full-time student

☐ Part-time student

☐ Retired

☐ Unable to work

13. In your household, what is the total approximate yearly income?

- ☐ Less than £12,750
- ☐ £12,571 - £37,500
- ☐ £37,501 - £59,999
- ☐ £60,000 - £99,999
- ☐ £100,000+
- ☐ Prefer not to say

14. How many people usually live at your permanent home address?

15. How many bathrooms do you have in your home?

16. In your household, how often do you do the cooking?

- ☐ Never
- ☐ 1-2 times a week
- ☐ 3-4 times a week
- ☐ 5-6 times a week
- ☐ Everyday

17. In your household, how often do you eat with others?

- ☐ Never
- ☐ Sometimes
- ☐ About half the time
- ☐ Most of the time
- ☐ Always

18. What county do you live in? (e.g., Dorset, Tyne and Wear).

Thank you very much for taking part. If there are any other comments you would like to make about sustainable eating from this questionnaire, please make those comments here:

Intake Frequency Estimates

Frequencies of food intakes are reported in Table S1. Fruits and vegetables were the most frequently consumed category (26.29 servings/week), with higher intake among females (26.90 servings/week) than males (25 servings/week). Ruminant meat consumption was higher among males (3.36 servings/week) than females (2.61 servings/week). In contrast, offal and future foods were consumed at a very low frequency.

Table S1. Participants' current frequency of certain food items assuming a standard frequency throughout time (*N = 635*).

Food Category	General population	Male population	Female population
	Average frequency consumed per week		
Dairy	11.54	11.64	11.49
Eggs / Cheese	3.43	3.44	3.43
Non-dairy alternatives	3.05	2.95	3.11
Ruminant meat	2.85	3.36	2.61
Poultry	1.94	1.97	1.92
Offal	0.07	0.11	0.05
Fish	1.57	1.48	1.61
Legumes	1.37	1.21	1.45
Future foods	0.06	0.05	0.06
Protein shakes	0.49	0.53	0.47
Fruits and vegetables	26.29	25.00	26.90
Bread items	5.01	4.93	5.04

Table S2. Means, standard deviations, and correlation coefficients of what people are currently doing versus their actual intakes.

Variables	Mean Frequency	Std Dev	Pearson Correlation
1. Swap white foods for wholegrain foods	2.16	2.43	0.377**
**Intake score** = (wholegrain total – white total)	1.52	4.16	
2. Swap any beef, lamb, or pork intake for chicken	1.24	1.88	0.240**
**Intake score** = (total chicken – total beef, lamb, and pork intake)	1.54	1.75	
3. Swap all meat intake for pulses	1.20	2.27	0.376**
**Intake score** = (total pulses – total meat intake)	-1.54	1.75	
4. Swap portions of meat for 'future food' substitutes	0.17	0.91	-0.039
**Intake score** = (total future foods – total meat intake)	1.54	1.75	
5. Swap any beef, lamb, or pork intake for offal	0.14	0.73	-0.021
**Intake score** = (total offal – total beef, lamb, and pork intake)	-0.35	0.59	

Note: * Correlation is significant at the 0.05 level (2-tailed). ** Correlation is significant at the 0.01 level (2-tailed). - = minus

Table S3. Results of all regression analyses for the sustainable actions the UK population currently do (N = 635)

	**Drink the recommended amount of water per day**		**Ensure you have no leftovers**		**Use your freezer more**		**Only snack on fruits, veg, and nuts**		**Replace white foods for wholegrain foods**		**Ensure all meat is farmed in UK**		**Drink Fairtrade tea/coffee**		**Have two days with no meat consumption**		**Ensure all vegetables in your diet are seasonal**	
Regression Equations for the Final Models	$R = 0.37$, $R^2 = 0.13$, adj. $R^2 = 0.12$, $F(13,632) = 7.60$, $p < 0.001$		$R = 0.42$, $R^2 = 0.18$, adj. $R^2 = 0.16$, $F(13,632) = 10.56$, $p < 0.001$		$R = 0.27$, $R^2 = 0.07$, adj. $R^2 = 0.05$, $F(13,632) = 3.98$, $p < 0.001$		$R = 0.30$, $R^2 = 0.09$, adj. $R^2 = 0.07$, $F(13,632) = 4.96$, $p < 0.001$		$R = 0.32$, $R^2 = 0.10$, adj. $R^2 = 0.08$, $F(13,632) = 5.46$, $p < 0.001$		$R = 0.24$, $R^2 = 0.05$, adj. $R^2 = 0.03$, $F(13,632) = 2.93$, $p < 0.001$		$R = 0.38$, $R^2 = 0.14$, adj. $R^2 = 0.12$, $F(13, 632) = 2.42$, $p < 0.001$		$R = 0.39$, $R^2 = 0.15$, adj. $R^2 = 0.14$, $F(13,632) = 8.94$, $p < 0.001$		$R = 0.35$, $R^2 = 0.12$, adj. $R^2 = 0.11$, $F(13,632) = 6.98$, $p < 0.001$	
	**Beta**	***p***	**Beta**	***p***	**Beta**	***p***	**Beta**	***p***	**Beta**	***p***	**Beta**	***p***	**Beta**	***p***	**Beta**	***p***	**Beta**	***p***
Gender (male/female)	0.234	0.21	0.204	0.26	0.141	0.52	**-0.709**	**0.002**	0.263	0.19	-0.081	0.28	-0.084	0.69	0.230	0.25	0.106	0.47
Age (years)	**-0.147**	**0.01**	**0.140**	**0.01**	-0.007	0.91	0.116	0.08	0.021	0.73	**-0.067**	**0.003**	-0.099	0.12	0.096	0.10	-0.043	0.33
Employment (status)	-0.039	0.23	0.010	0.75	0.024	0.53	0.003	0.93	-0.057	0.10	0.010	0.46	**-0.077**	**0.03**	0.007	0.84	0.022	0.40
Education (level)	**0.143**	**0.009**	0.064	0.21	**-0.138**	**0.03**	0.013	0.84	0.043	0.46	0.009	0.69	**0.126**	**0.04**	**0.121**	**0.03**	0.047	0.27
Income	-0.085	0.31	-0.024	0.76	-0.003	0.97	-0.022	0.83	-0.104	0.25	-0.018	0.59	0.081	0.40	0.027	0.76	0.040	0.54
Socio-economic status	-0.164	0.36	0.013	0.93	-0.058	0.78	-0.007	0.97	0.171	0.38	-0.008	0.90	**-0.411**	**0.04**	0.221	0.25	-0.126	0.38
Food cooking	**0.097**	**0.02**	0.014	0.73	0.050	0.33	0.084	0.11	0.014	0.77	0.025	0.16	0.080	0.11	**0.086**	**0.06**	-0.030	0.38
Mealtimes with others	0.039	0.39	-0.012	0.78	-0.030	0.57	-0.003	0.96	-0.079	0.11	0.000	0.99	**-0.098**	**0.06**	**-0.110**	**0.02**	-0.039	0.27
Knowledge	-0.094	0.48	**0.275**	**0.03**	**0.479**	**0.003**	0.146	0.37	0.215	0.14	0.030	0.58	**0.726**	**<.001**	0.209	0.14	0.087	0.41
Impact	**0.595**	**<.001**	0.216	0.15	0.004	0.98	0.317	0.10	**0.370**	**0.03**	-0.055	0.39	0.036	0.84	0.386	0.02	**0.293**	**0.02**
Perceived importance	**0.892**	**<.001**	**1.401**	**<.001**	**0.847**	**<.001**	**1.160**	**<.001**	**0.900**	**<.001**	**0.226**	**0.004**	**0.687**	**0.002**	**1.096**	**<.001**	**0.995**	**<.001**
Small changes	**-0.282**	**0.05**	0.008	0.95	**0.365**	**0.03**	-0.188	0.28	0.064	0.68	**0.154**	**0.009**	-0.247	0.13	0.070	0.65	-0.161	0.16
Perceived value	-0.004	0.97	0.103	0.44	-0.035	0.83	-0.249	0.14	-0.044	0.77	0.089	0.11	**0.342**	**0.03**	0.106	0.47	0.028	0.79

	Eat the whole vegetable		Only eat fruits/veg that have ethical trade stamps		Replace ruminant meat for chicken		Replace all meat items for pulses		Buy all fruit and vegetables from local shops		Only eat organic fruits and vegetables		Eat fish that are low in food chain		Replace meat items for future foods		Swap ruminant meat for offal	
Regression Equations for the Final Models	R = 0.35, R^2 = 0.12, adj. R^2 = 0.11, F(13,632) = 6.99, p < 0.001		R = 0.21, R^2 = 0.04, adj. R^2 = 0.02, F(13,632) = 2.35, p < 0.001		R = 0.24, R^2 = 0.06, adj. R^2 = 0.04, F(13,632) = 3.01, p < 0.001		R = 0.25, R^2 = 0.06, adj. R^2 = 0.04, F(13, 632) = 3.20, p < 0.001		R = 0.33, R^2 = 0.11, adj. R^2 = 0.09, F(13,632) = 6.03, p < 0.001		R = 0.34, R^2 = 0.11, adj. R^2 = 0.09, F(13,632) = 6.23, p < 0.001		R = 0.39, R^2 = 0.15, adj. R^2 = 0.13, F(13,632) = 8.75, p < 0.001		R = 0.36, R^2 = 0.13, adj. R^2 = 0.11, F(13,632) = 7.41, p < 0.001		R = 0.22, R^2 = 0.04, adj. R^2 = 0.29, F(13,632) = 2.46, p < 0.001	
	**Beta**	***p***	**Beta**	***p***	**Beta**	***p***	**Beta**	***p***	**Beta**	***p***	**Beta**	***p***	**Beta**	***p***	**Beta**	***p***	**Beta**	***p***
Gender (male/female)	0.389	0.10	-0.159	0.12	-0.059	0.72	0.263	0.12	0.248	0.27	0.070	0.71	0.083	0.61	**0.536**	**0.01**	-0.109	0.08
Age (years)	-0.116	0.10	0.054	0.08	-0.091	0.06	-0.028	0.58	-0.018	0.79	0.008	0.88	**-0.137**	**0.006**	-0.051	0.40	-0.014	0.45
Employment (status)	-0.061	0.14	-0.010	0.56	0.021	0.45	0.015	0.61	-0.051	0.19	0.007	0.84	-0.049	0.09	-0.043	0.23	0.003	0.75
Education (level)	**0.235**	**<.001**	**0.057**	**0.05**	**-0.099**	**0.03**	0.011	0.81	-0.029	0.66	0.028	0.61	0.045	0.34	**0.125**	**0.03**	-0.003	0.86
Income	0.052	0.62	-0.007	0.88	0.052	0.47	-0.012	0.87	0.020	0.84	0.018	0.83	0.067	0.36	-0.151	0.10	0.004	0.88
Socio-economic status	-0.259	0.26	0.087	0.38	0.220	0.16	-0.031	0.85	0.191	0.38	0.059	0.74	-0.137	0.39	-0.151	0.45	0.112	0.06
Food cooking	0.026	0.64	-0.020	0.42	0.056	0.14	0.019	0.63	0.075	0.16	**0.094**	**0.03**	-0.067	0.08	-0.013	0.78	0.000	0.98
Mealtimes with others	0.003	0.96	0.022	0.37	-0.019	0.63	-0.003	0.93	0.054	0.33	0.028	0.54	-0.063	0.11	0.041	0.41	0.001	0.95
Knowledge	0.178	0.30	0.115	0.12	0.015	0.89	**0.270**	**0.02**	**0.406**	**0.01**	**0.493**	**<.001**	**0.238**	**0.04**	-0.034	0.81	0.004	0.93
Impact	-0.154	0.44	-0.087	0.31	0.020	0.88	-0.004	0.97	0.256	0.18	0.290	0.07	0.257	0.06	**0.571**	**<.001**	-0.063	0.23
Perceived importance	**1.183**	**<.001**	**0.200**	**0.05**	**0.707**	**<.001**	**0.586**	**<.001**	**1.181**	**<.001**	**0.540**	**0.006**	**0.995**	**<.001**	**1.107**	**<.001**	**0.218**	**<.001**
Small changes	0.297	0.11	**0.169**	**0.03**	0.138	0.27	0.048	0.71	-0.124	0.48	0.057	0.70	-0.026	0.84	-0.282	0.07	**0.124**	**0.01**
Perceived value	**0.511**	**0.004**	**0.150**	**0.05**	0.113	0.35	0.185	0.14	-0.105	0.53	0.121	0.39	0.152	0.21	0.066	0.66	0.053	0.24

Table S4. Results of all regression analyses for the sustainable actions the UK population sample are not currently doing but are willing to adopt (the composite NCU-W score) (N= 635).

	**S1: Replace all meat items for pulses**		**S2: Ensure all vegetables in your diet are seasonal**		**S3: Ensure all meat is farmed in UK**		**S4: Ensure you have no leftovers**		**S5: Eat the whole vegetable**		**S6: Replace meat items for future foods**		**S7: Drink the recommended water**		**S8: Replace white foods for wholegrain foods**		**S9: Only eat organic fruits and vegetables**	
Regression Equations for the Final Models	R = 0.40, R^2 = 0.16, adj. R^2 = 0.14, F(13, 632) = 9.52, p < 0.001		R = 0.28, R^2 = 0.08, adj. R^2 = 0.06, F(13,632) = 4.25, p < 0.001		R = 0.28, R^2 = 0.08, adj. R^2 = 0.06, F(13,632) = 04.26, p < 0.001		R = 0.21, R^2 = 0.04, adj. R^2 = 0.02, F(13,632) = 2.33, p < 0.001		R = 0.31, R^2 = 0.10, adj. R^2 = 0.08, F(13,632) = 5.35, p < 0.001		R = 0.29, R^2 = 0.08, adj. R^2 = 0.06, F(13,632) = 4.54, p < 0.001		R = 0.25, R^2 = 0.06, adj. R^2 = 0.04, F(13,632) = 3.42, p < 0.001		R = 0.32, R^2 = 0.10, adj. R^2 = 0.08, F(13,632) = 5.50, p < 0.001		R = 0.39, R^2 = 0.15, adj. R^2 = 0.14, F(13,632) = 9.02, p < 0.001	
	**Beta**	***p***	**Beta**	***p***	**Beta**	***p***	**Beta**	***p***	**Beta**	***p***	**Beta**	***P***	**Beta**	***p***	**Beta**	***p***	**Beta**	***p***
Gender (male/female)	0.996	0.15	0.050	0.92	0.164	0.76	0.113	0.79	-0.218	0.72	-0.191	0.78	-0.641	0.11	0.580	0.29	0.974	0.13
Age (years)	0.038	0.85	-0.204	0.20	-0.043	0.79	**-0.371**	**0.005**	**-0.493**	**0.007**	**-0.604**	**0.005**	**-0.242**	**0.04**	**-0.484**	**0.003**	**-0.506**	**0.009**
Employment (status)	-0.152	0.21	0.067	0.47	0.055	0.56	-0.089	0.24	-0.125	0.24	0.116	0.35	0.025	0.72	-0.059	0.54	0.019	0.86
Education (level)	**0.510**	**0.01**	0.089	0.56	-0.050	0.74	-0.061	0.63	0.237	0.18	**0.577**	**0.005**	**-0.256**	**0.03**	0.054	0.73	0.057	0.75
Income	-0.114	0.71	0.274	0.25	-0.035	0.88	0.139	0.48	0.191	0.48	**-0.678**	**0.03**	**0.405**	**0.02**	-0.269	0.27	0.294	0.31
Socio-economic status	-0.910	0.17	-0.875	0.09	-0.737	0.15	0.613	0.15	-0.192	0.74	0.089	0.89	-0.064	0.87	-0.289	0.58	-0.743	0.23
Food cooking	-0.133	0.42	0.150	0.23	0.107	0.40	**-0.181**	**0.08**	0.018	0.89	-0.111	0.50	-0.270	0.005	**-0.280**	**0.03**	-0.056	0.71
Mealtimes with others	0.219	0.20	0.024	0.85	0.068	0.60	0.052	0.63	-0.118	0.43	0.098	0.57	0.101	0.30	0.040	0.76	-0.079	0.61
Knowledge	-0.560	0.27	-0.243	0.53	0.406	0.29	-0.408	0.20	0.403	0.36	-0.503	0.32	-0.358	0.22	0.036	0.92	**1.072**	**0.02**
Impact	-0.070	0.90	0.507	0.26	0.052	0.90	0.315	0.39	-0.217	0.67	0.162	0.78	-0.129	0.70	**0.989**	**0.03**	-0.094	0.86
Perceived importance	**6.836**	**<.001**	**2.886**	**<.001**	**2.710**	**<.001**	**1.208**	**0.008**	**3.854**	**<.001**	**3.414**	**<.001**	0.174	0.67	**2.757**	**<.001**	**4.942**	**<.001**
Small changes	-0.664	0.22	-0.157	0.70	**-1.048**	**0.01**	-0.314	0.35	-0.472	0.32	**-1.413**	**0.01**	0.032	0.91	-0.550	0.19	**-1.549**	**0.002**
Perceived value	-0.494	0.34	0.601	0.13	**0.861**	**0.03**	-0.272	0.40	0.338	0.45	**1.006**	**0.05**	-0.031	0.91	0.260	0.52	0.695	0.14

	**S10: Drink Fairtrade tea/coffee**		**S11: Eat fish that are low in food chain**		**S12: Replace ruminant meat for chicken**		**S13: Buy all fruit and vegetables from local shops**		**S14: Use your freezer more**		**S15: Only snack on fruits, veg, and nuts**		**S16: Only eat fruits/veg that have ethical trade stamps**		**S17: Have no meat consumption**		**S18: Swap ruminant meat for offal**	
Regression Equations for the Final Models	R = 0.38, R^2 = 0.15, adj. R^2 = 0.13, F(13, 632) = 8.51, p < 0.001		R = 0.32, R^2 = 0.10, adj. R^2 = 0.08, F(13,632) = 5.68, p < 0.001		R = 0.24, R^2 = 0.06, adj. R^2 = 0.04, F(13,632) = 3.10, p < 0.001		R = 0.37, R^2 = 0.14, adj. R^2 = 0.12, F(13,632) = 7.76, p < 0.001		R = 0.16, R^2 = 0.02, adj. R^2 = 0.05, F(13,632) = 1.25, p < 0.001		R = 0.28, R^2 = 0.07, adj. R^2 = 0.05, F(13,632) = 4.07, p < 0.001		R = 0.39, R^2 = 0.15, adj. R^2 = 0.13, F(13,632) = 8.64, p < 0.001		R = 0.31, R^2 = 0.09, adj. R^2 = 0.07, F(13,632) = 5.14, p < 0.001		R = 0.26, R^2 = 0.06, adj. R^2 = 0.04, F(13,632) = 3.50, p < 0.001	
	**Beta**	***p***	**Beta**	***p***	**Beta**	***P***	**Beta**	***p***	**Beta**	***p***	**Beta**	***p***	**Beta**	***p***	**Beta**	***p***	**Beta**	***p***
Gender (male/female)	0.367	0.55	-0.189	0.79	-0.674	0.26	1.077	0.09	-0.245	0.59	**1.584**	**0.004**	0.718	0.26	0.099	0.87	**-1.814**	**0.01**
Age (years)	**-0.460**	**0.01**	**0.746**	**<.001**	-0.247	0.17	0.147	0.43	-0.017	0.89	-0.251	0.12	**-0.419**	**0.02**	0.015	0.93	**0.512**	**0.01**
Employment (status)	0.084	0.43	-0.063	0.62	-0.024	0.81	-0.055	0.62	-0.071	0.36	0.001	0.99	-0.162	0.14	0.026	0.81	0.033	0.79
Education (level)	0.018	0.91	0.365	0.08	0.084	0.63	0.070	0.70	-0.083	0.52	-0.005	0.97	-0.005	0.97	-0.069	0.70	**0.507**	**0.01**
Income	0.188	0.49	0.047	0.88	0.278	0.30	-0.016	0.95	-0.011	0.95	0.219	0.36	0.073	0.79	0.078	0.78	-0.373	0.24
Socio-economic status	-0.312	0.59	0.744	0.29	-0.063	0.91	-0.895	0.14	-0.778	0.07	-0.738	0.15	**-1.279**	**0.03**	-0.444	0.47	-0.506	0.46
Food cooking	-0.017	0.90	**-0.513**	**0.003**	-0.102	0.47	-0.092	0.53	-0.136	0.20	0.157	0.21	0.069	0.64	0.143	0.34	**-0.335**	**0.04**
Mealtimes with others	0.105	0.48	-0.122	0.49	-0.112	0.44	0.175	0.25	0.020	0.85	-0.181	0.16	0.146	0.34	0.255	0.10	-0.067	0.70
Knowledge	0.720	0.10	0.408	0.43	**-1.052**	**0.01**	0.110	0.81	-0.202	0.53	**0.757**	**0.05**	-0.069	0.88	**-1.163**	**0.01**	-0.414	0.42
Impact	0.549	0.28	-0.661	0.28	-0.072	0.88	0.028	0.95	0.377	0.32	0.449	0.32	-0.008	0.98	0.133	0.80	-0.721	0.23
Perceived importance	**4.421**	**<.001**	**4.776**	**<.001**	**3.157**	**<.001**	**4.225**	**<.001**	0.607	0.19	**1.507**	**0.007**	**4.959**	**<.001**	**4.829**	**<.001**	**2.747**	**<.001**
Small changes	**-1.347**	**0.005**	-0.248	0.65	-0.097	0.83	-0.540	0.26	0.551	0.11	-0.574	0.17	**-1.159**	**0.01**	-0.753	0.12	0.392	0.48
Perceived value	0.798	0.07	0.540	0.68	0.002	0.99	**1.974**	**<.001**	-0.439	0.19	0.149	0.71	**1.103**	**0.01**	-0.033	0.94	0.494	0.35
